# Alcoholic Liquors in the Practice of Medicine

**Published:** 1887-07

**Authors:** 


### ALCOHOLIC LIQUORS IN THE PRACTICE OF MEDICINE.

We begin in this issue of THE JOURNAL an interesting and well-written paper on this subject by Dr. Eugene Foster, of Augusta. It is a review of the paper of Dr. Joseph P. Logan, which was read at the meeting of the Medical Association of Georgia in 1886, and which appeared in the July number of THE JOURNAL for that year.

Dr. Foster's paper was read at the recent meeting of the Association in this city, and by resolution was ordered published in THE JOURNAL.

It will be remembered by those of our readers who read Dr. Logan's paper that he took the ground that only in rare cases, if ever at all, was alcoholic liquor beneficial as a medicine.

Dr. Foster takes the position that there is a well-defined place for alcoholic liquors in the materia medica, and makes a strong argument in their favor.

### ITEMS.

DR. W. S. ELKIN is spending a few weeks at his old home in Kentucky.

DR. W. F. WESTMORELAND, JR., has returned from a three-months visit to New York.

DR. J. S. TODD, of this city, is spending a short vacation at the sea-shore on St. Simon's Island.

DR. W. S. KENDRICK has located in Atlanta. He is occupying the office and home of Dr. J. P. Logan, on Washington street.

DR. JOHN THAD. JOHNSON, after spending two years in California, has returned to Atlanta and resumed the practice of medicine.

Dr. A. G. WHITEHEAD, of Waynesboro, Ga., President of the Medical Association of Georgia, is on a visit to San Francisco, California.

DR. J. P. LOGAN, of this city, was married on June 23d to Miss Alice Clarke, sister of Hon. Marshall J. Clarke. They will sail from New York on July 2d for Europe, and will be absent until October.

THE American Medical Association has voted that hereafter there shall be a dinner provided as one of the entertainments at the meetings, and that members who attend shall pay a fixed fee, which shall be different for those who do and those who do not drink wine.

COLORLESS TINCTURE OF IODINE.—Dr. A. J. Brewer, of Mountain Home, Arkansas, writes: "I find a formula in the fifteenth edition of U. S. D. for making colorless tincture of iodine. Equal parts of compound tincture of iodine and aqua ammonia mixed constitute the formula. This must stand for twenty-four hours before it becomes colorless. I find by adding four drops of carbolic acid to the ounce, and shaking, it becomes colorless instantly."

MR. GEORGE S. DAVIS, Medical Publisher, Detroit, Michigan, will continue the publication of the "Physicians' Leisure Library," begun last year. The series for 1887 begin with the diagnosis and treatment of hæmorrhoids, by Dr. Chas. B. Kelsey. This volume was issued June 1st. It will be followed by diseases of the heart, vol. 1, by Dujarden–Beaumetz, issued July 1st. The entire series is made up of practical works by the best writers in

America and Europe, and will be issued from time to time during the year, the last one will be delivered December 16th. The entire series for 1886 or 1887 will be delivered for $2.50, single copies 25 cents. Never before have physicians been offered such valuable works for so little money.

A PHYSICIAN of twenty years experience, a graduate of Bellview Medical College, desirous of getting into a milder climate, would be glad to associate himself with some institution, infirmary, or physician of large practice, or one that may want to retire soon to do a special practice, rectal diseases, gynæcology, or genito-urinary diseases, in one of the large cities South, Georgia or Tennessee, prefered. Best of references given and required. Address, Specialty Company, Matteawan, New York, Box 435.

OFFICERS AMERICAN MEDICAL ASSOCIATION.—President, A. Y. P. Garnet, of Washington; First Vice-President, Duncan Eve, Nashville, Tenn.; Second Vice-President, David Collville, New York; Third Vice-President, Charles J. O. Hagan, North Carolina; Fourth Vice-President, A. Stedman, Colorado. Librarian, C. H. A. Kleinschmidt, Washington; Treasurer, R. J. Dunglison, Philadelphia; Permanent Secretary, W. B. Atkinson, Philadelphia; Assistant Secretary, Joseph Ransohoff, Cincinnati; Trustees of the Journal, Leartus Connor, Detroit; E. O. Shakespeare, Philadelphia; W. T. Briggs, Nashville; Judicial Council, J. H. Murphy, Minn.; J. M. Toner, Washington; J. K. Bartlett, Milwaukee; A. B. Sloane, Missouri; X. C. Scott, Ohio; A. W. McLuer, Iowa; D. W. Stormont, Kansas; J. H. Hibberd, Indiana; Committee on Necrology, J. M. Toner, Chairman; Committee on State Medicine, R. G. Jennings, Arkansas, Chairman. The next annual meeting is to be held at Cincinnati on the second Tuesday in May, 1888. Chairman of Committee of Arrangements, Dr. W. W. Dawson, of Cincinnati, with power to fill the Committee by appointment.

